# Prenatal exposure to poly-/per-fluoroalkyl substances is associated with alteration of lipid profiles in cord-blood

**DOI:** 10.1007/s11306-021-01853-9

**Published:** 2021-11-24

**Authors:** Lisanna Sinisalu, Leo W. Y. Yeung, Jinghua Wang, Yitao Pan, Jiayin Dai, Tuulia Hyötyläinen

**Affiliations:** 1 School of Science and Technology, Örebro University, 702 81 Örebro, Sweden; 2 Key Laboratory of Animal Ecology and Conservation Biology, Institute of Zoology, Chinese Academy of Sciences, Beijing, 100101 China

**Keywords:** Prenatal exposure, Bile acids, Lipidomics, Mass spectrometry, PFAS, Cord blood

## Abstract

**Introduction:**

Poly-/per-fluoroalkyl substances (PFAS) are widespread environmental pollutants that may induce metabolic perturbations in humans, including particularly alterations in lipid profiles. Prenatal exposure to PFAS can cause lasting effects on offspring metabolic health, however, the underlying mechanisms are still unknown.

**Objectives:**

The goal of the study was to investigate the impact of prenatal PFAS exposure on the lipid profiles in cord blood.

**Methods:**

Herein, we combined determination of bile acids (BAs) and molecular lipids by liquid chromatography with ultra-high-resolution mass spectrometry, and separately quantified cord blood concentrations of sixteen PFAS in a cohort of Chinese infants (104 subjects) in a cross-sectional study. We then evaluated associations between PFAS concentration and lipidome using partial correlation network analysis, debiased sparse partial correlation, linear regression analysis and correlation analysis.

**Results:**

PFAS levels showed significant associations with the lipid profiles; specifically, PFAS exposure was positively correlated with triacylgycerols (TG) and several bile acids. Importantly, exposure to perfluorooctanoic acid (PFOA), perfluorooctane sulfonic acid (PFOS), perfluorononanoic acid (PFNA) and perfluorohexane sulfonic acid (PFHxS) were associated with increased levels of TGs with saturated fatty acids while multiple classes of phospholipids were decreased. In addition, several free fatty acids showed significant positive correlations with PFOS.

**Conclusions:**

Our results indicated that prenatal exposure to PFAS mediated metabolic changes, which may explain the associations reported between PFAS exposure and metabolic health later in life.

**Supplementary Information:**

The online version contains supplementary material available at 10.1007/s11306-021-01853-9.

## Introduction

Poly-/Per-fluoroalkyl substances (PFAS) are a class of persistent environmental chemicals which are used in many industrial applications and consumer products, including cookware, clothing, food packaging, cleaning products, paints, and fire-fighting foams. There is a widespread exposure in the general population, mainly through diet and contaminated water, with children being additionally exposed to PFAS during the prenatal period via transplacental transfer and during infancy via breastfeeding (Haug et al., 2011). Particularly, children are vulnerable to PFAS exposure as they are more heavily exposed in proportion to their body weight, and they are going through sensitive windows for development. PFAS exposure has been identified to be linked with a variety of adverse health outcomes in both human epidemiology and animal toxicology studies (Alderete et al., 2019; Bijland et al., 2011a; Cardenas et al., 2017; Jin et al., 2020; Schlezinger et al., 2020, 2021; Sun et al., 2018) including positive associations between PFAS exposure and dyslipidemia, immunity, renal function and age at menarche (Rappazzo et al., 2017), autoimmune diseases (McGlinchey et al., 2019; Sinisalu et al., 2020), with cardiometabolic risk factors, reduced birth weight, reduced birth length, and increased adiposity as well as with higher insulin levels (Halldorsson et al., 2012).

The exact mechanisms behind the adverse health impacts are not fully understood. Given the structural similarity of PFAS with fatty acids, PFAS may induce metabolic alterations. Indeed, many of the reported risk factors have been linked with alterations in the metabolome. Animal studies have indicated changes on fatty acid beta-oxidation as well as in bile acid metabolism (Das et al., 2017; Geng et al., 2019; Zhao et al., 2015). At organism level, it has been suggested that exposure to PFAS increases steatosis, i.e. hepatic accumulation of lipids (Christensen et al., 2019; Sunderland et al., 2019). We have also previously shown that prenatal and early life PFAS exposure is associated with changes in lipid profiles, particularly in specific phospholipids and triglycerides (McGlinchey et al., 2019; Salihović et al., 2019), suggesting that the lipid changes may be modulated via changes in bile acids (Zhao et al., 2016). Bile acids are not only facilitating the digestion and absorption of larger lipids in the small intestine but are also important metabolic regulators involved in the maintenance of lipid and glucose homeostasis (Haeusler et al., 2013; Prawitt et al., 2011). PFAS have shown to disturb the bile acid metabolism at several levels. In the liver, PFAS have been shown to down-regulate the cholesterol 7a-hydroxylase (Cyp7A1), which catalyzes the first and rate-limiting step in the formation of bile acids from cholesterol through the main, classic pathway (Beggs et al., 2016; Chiang, 2017). PFAS also inhibit the function of the hepatocyte nuclear factor 4α (Buhrke et al., 2015), which plays a central role in the regulation of bile acids and is linked both with the synthesis and conjugation of primary bile acids. PFAS exposure has also been shown to modulate the composition of the gut microbiota (Cheng et al., 2018; Iszatt et al., 2019; Pan et al., 2019; Petriello et al., 2018; Rude et al., 2019) which in turn can cause alteration in the pool of secondary bile acids. Moreover, PFAS are also using the same transporter system that transports the BAs from the portal vein back to liver (Zhao et al., 2015).

Herein, we hypothesized that exposure to PFAS impact lipid metabolism, specifically associated with changes in bile acid metabolism. We measured the levels of PFAS, lipids and bile acids in cord plasma samples from Beijing, China (*n* = 104) and investigated the associations between bile acids, lipid and PFAS levels.

## Methods

### Study population

A total number of 104 cord plasma samples collected from 2018 were archived in the Beijing Cord Blood Bank (Beijing, China) (Wang et al., 2020). Shortly, the cord blood sample was collected into a plastic blood bag containing citrate phosphate dextrose adenine as an anticoagulant, and then was shipped to Beijing Cord Blood Bank within 24 h. Blood sample was centrifuged (1500×*g* for 20 min, 4 ℃), and the plasma was stored in cryogenic vial at − 40 ℃. The plasma samples were delivered to the laboratory on dry ice and kept in − 80 ℃ until PFASs analysis. Details of the sample collection can be found at: http://www.globalcordbloodcorp.com/en_US/. Concentrations of PFAS were measured in another study (Wang et al., 2020). Clinical characteristics such as maternal age, delivery type, and infant gender and birth weight were obtained from obstetric records are provided in Table 1. The plasma samples were stored at − 80 °C until further analysis.

**Table 1:** Demographics of the subjects

	Mean ± SD or n (%)
Age at delivery (year)	28 ± 2
< 25	10 (9.9)
25–29	78 (77.2)
> 29	13 (12.9)
Type of delivery	
Vaginal delivery	57 (56.4)
C-section	44 (43.6)
Gender	
Male	50 (49.5)
Female	51 (50.5)
Birth weight (g)	3458 ± 381
2500–4499	100 (99.0)
> 4499	1 (1.0)

## Materials

PFAS standards were purchased from Wellington Laboratories (Guelph, ON, Canada), except for 4:2 Cl-PFESA and 6:2 H-PFESA, which were synthesized by Dr. Yong Guo at the Shanghai Institute of Organic Chemistry (Chinese Academy of Sciences). Ammonium acetate (≥ 99.9%), tetrabutylammonium hydrogen sulfate (TBAS, ≥ 97%), sodium carbonate (≥ 99.5%), and sodium bicarbonate (≥ 99.7%) were purchased from Sigma-Aldrich (St. Louis, MO, USA). LC–MS grade methanol (MeOH), methyl-*tert*-butyl ether (MTBE), formic acid, and water were purchased from Fisher Scientific (Pittsburgh, PA, USA). Chloroform A.R. (CHCl_3_, with 0.3–1.0% ethanol (C_2_H_5_OH) from Beijing Chemical Works (Beijing, China) and HPLC Chloroform (99.9%) from Sigma-Aldrich (Saint Louis, Missouri, the United States of America). Fetal bovine serum (FBS) was obtained from Gibco (Paisley, UK) as quality control samples. A standard reference material SRM1957—Organic Contaminants in Non-Fortified Human Serum was obtained from the National Institute of Standards and Technology (NIST, USA). Quality control (QC) pooled human blood samples were from donors from Örebro University Hospital, Sweden. The bile acid standards and lipid standards and their origin, together with their abbreviations are listed in Supplementary Table 1.

## PFAS analysis

### Sample extraction

PFAS in cord plasma sample was extracted using an ion-pair method with modifications (Wang et al., 2018). Briefly, 200 μL of sample was spiked with 0.5 ng of internal standards and extracted with 1 mL of TBAS (0.5 mol/L), 2 mL of NaHCO_3_/Na_2_CO_3_ (pH = 10), and 4 mL of MTBE. The organic phase was collected after mixing at 300 rpm for 30 min (at room temperature) and centrifugation at 4400 rpm for 15 min and the aqueous phase was re-extracted. The combined extracts were spiked with 0.5 ng of mass-labelled recovery standard, evaporated to dryness and reconstituted with 200 μL of methanol.

### Instrumental analysis

PFAS were analyzed using ultra-performance liquid chromatography-tandem mass spectrometry (UPLC-MS/MS) on a Waters Acquity UPLC system interfaced with a Xevo triple quadrupole mass spectrometer (Waters, Milford, MA, USA) operated in electrospray-negative ionization mode as described before (Wang et al., 2018).

### Quality assurance and quality control

Quantification of PFAS was performed using a 12-point internal calibration curve (0.01–20 ng/mL). All curves exhibited linearity with R^2^ > 0.99. Matrix spike recoveries were assessed by spiking known amounts of mixed native standards (having final concentrations at 0.1, 1, 10 ng/mL) into a surrogate FBS matrix to check the reliability of the method; recoveries ranged from 75.5% to 111%. In each batch, one extraction blank and one SRM1957 sample were extracted with every 20 samples to evaluate background contamination, instrumental performance, and possible interferences. No detectable PFASs were found in extraction blanks.

## Lipidomic and bile acid analyses

### Sample extraction

After randomization of the samples 40 µL of plasma sample was extracted with 270 µL of MeOH/MTBE/CHCl_3_ (1.33:1:1, v/v/v) after addition of 10 µL of BA + PFAS internal standard mixture (c = 200 ng/mL PFAS and 1000 ng/mL bile acids in acetonitrile) and 12 µL of lipid internal standard mixture (c = 25 µg/mL chloroform:methanol, 2:1 v/v). The samples were then incubated on a shaker at 900 rpm for 1 h and centrifuged (9600 RCF, 10 min). 250 µL of the supernatant was collected and evaporated to dryness. For lipidomic analyses, the samples were dissolved into 200 µL of CHCl_3_/MeOH (2:1). After lipidomic analyses, the samples were evaporated to dryness and reconstituted with 40 µL of MeOH/H_2_O (70%/30%, v/v).

### Instrumental analysis

The lipidomic analyses were done using an UHPLC- quadrupole time-of-flight mass spectrometry method (Agilent Technologies, Santa Clara, CA, USA) as described in (McGlinchey et al., 2019). Internal standard mixture was used for normalization and lipid-class specific calibration was used for quantitation as previously described (Sen et al., 2019). MS data processing was performed using open source software MZmine 2.53 (Pluskal et al., 2010). Bile acids were analyzed on a UHPLC-qTOF/MS (Agilent Technologies, Santa Clara, CA, The United States of America) with Acquity UPLC®, BEH C18 (2.1 × 100 mm, 1.7 µm) (Waters, Milford, MA, USA) column set at 50 °C with a C18 pre-column (Waters, Wexford, Ireland). The samples were kept at 10 °C and 10 µL of the sample volume was injected. The mobile phases were A: 2 mM NH_4_Ac in H_2_O:MeOH (70:30) and B: 2 mM NH_4_Ac in MeOH. The flow rate was 0.4 mL/min and the gradient started with 95% A and 5% B with a linear change after 1.5 min to 70% A and 30% B, followed a change after 4.5 min to 30% A and 70% B, and after 7.5 min with 100% B until the end of run. Dual jet stream electrospray (dual ESI) ion source was used in negative mode. The capillary voltage and the nozzle voltage were at 4500 V and 1500 V. The N_2_ pressure was set on 21 psi, with the sheath gas flow as 11 L/min and temperature at 379 °C for the nebulizer. The data was acquired with MassHunter B.06.01 software (Agilent Technologies, Santa Clara, CA, USA). MS data processing was performed using open source software MZmine 2.53 (Pluskal et al., 2010). Lipids were processed separately from BA data. The identification was done with a custom data base, with identification levels 1 and 2 identification, based on Metabolomics Standards Initiative (Sansone et al., 2007). However, it should be noted that the method cannot distinguish the isomers differing only by their double bond position.

### Quantitation and quality control

Quantification of lipids was performed using a 7-point internal calibration curve (0.1–5 µg/mL) and for bile acids using a 7-point calibration curve (0–360 µg/mL). Quality control was performed throughout the dataset both for lipidomics and bile acids by including blanks, pure standard samples, extracted standard samples and control plasma samples. The RSD of lipid concentrations in the pooled control samples (*n* = 5) and in the pooled serum samples (*n* = 9) was on average 15.8%. For bile acids, RSD concentrations in the pooled serum samples (*n* = 9) was on average 34.9%, with majority of the bile acids having RSD < 25%.

## Statistical analyses

Total PFAS values were calculated as a sum of all detectable individual PFAS. The subjects were divided into four exposure quartiles based on both the total PFAS concentrations as well as individual PFAS concentrations for those PFAS detected in > 70% of the samples (Supplementary Table 2). The metabolomic data was zero imputed, log_2_ transformed and autoscaled prior to the statistical analyses. PFAS results in this study with < LOD (limit of detection) values have been replaced with LOQ (limit of quantification) × 0.5. The statistical analyses were performed using SPSS Statistics, MetaboAnalyst 4 (Chong et al., 2019), and MetScape 3 for CytoScape (Basu et al., 2017). Debiased Sparse Partial Correlation algorithm (DSPC) was used for estimating partial correlation networks, visualised by the MetScape3 (Chong et al., 2019). A Spearman’s rank correlation analysis was used to assess correlation between PFAS in cord serum samples. Significance level was at p < 0.05. General linear regression model was used to analyze the associations between PFAS compounds and metabolites, with all the models were adjusted for maternal age, delivery type and birth weight as these factors showed association with the PFAS concentrations.

## Results

### Demographics and clinical characteristics of study population

Mean age of the mothers was 28 years. All infants were born full term, and newborn sex was approximately equally distributed. 57% of the newborns were born via vaginal delivery and 43% via C-section.

### PFAS and metabolome in cord blood

**Fig. 1:**
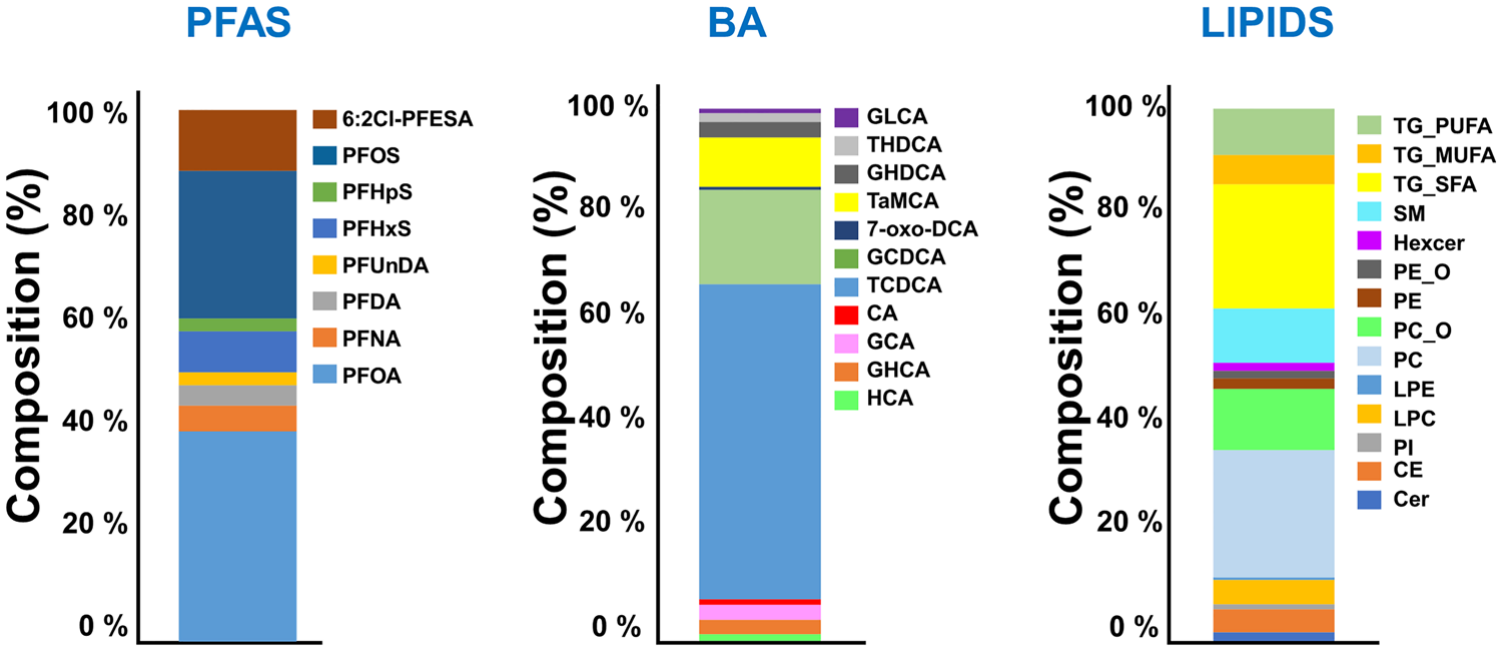
Composition (%) of PFAS, bile acids and lipids (as lipid classes) in cord plasma samples. PFAS with detection frequency over 15% is shown in the composition profile

A total of 14 out of 16 PFASs showed detectable concentrations in the cord plasma samples (Fig. 1, Supplementary table 2), with eight of them being present in > 15% of the samples. PFHxS, PFOS, PFOA and 6:2 Cl-PFESA were detected in > 99% of the samples while the longer chain PFAS were detected only in a few samples. The total PFAS concentrations varied between 0.68 and 5.4 ng/mL. There were statistically significant differences in total PFAS between female and male infants (*Mann–Whitney U test*, p < 0.05).

Eleven bile acids, including primary and conjugated bile acids as well as a few secondary bile acids were detected in the cord plasma/serum (Fig. 1). The two BA detected at highest concentration were the taurine and glycine conjugates of the primary bile acid CDCA. The levels of secondary bile acids (DCA, THDCA and GLCA) were very low, and this was also reflected in their higher analytical variation.

Two hundred and eighty seven lipids were identified, which covered phosphatidylcholine (PC), lysophostidylcholines (lysoPCs), phosphatidylethanolamines (PE), phosphatidylinositols (PI), ceramides (Cer), cholesterylesters (CE), sphingomyelins (SM), and di-and triacylglycerols (DG, TG). The main group of lipids were the PCs, alkyl ether PCs (PC_O) and TGs containing saturated fatty acyl chains (TG_SFA), and SMs. The identified lipids were further grouped into 14 lipid classes for data analyses (Fig. 1).

### Association with PFAS exposure on metabolic profiles

**Fig. 2:**
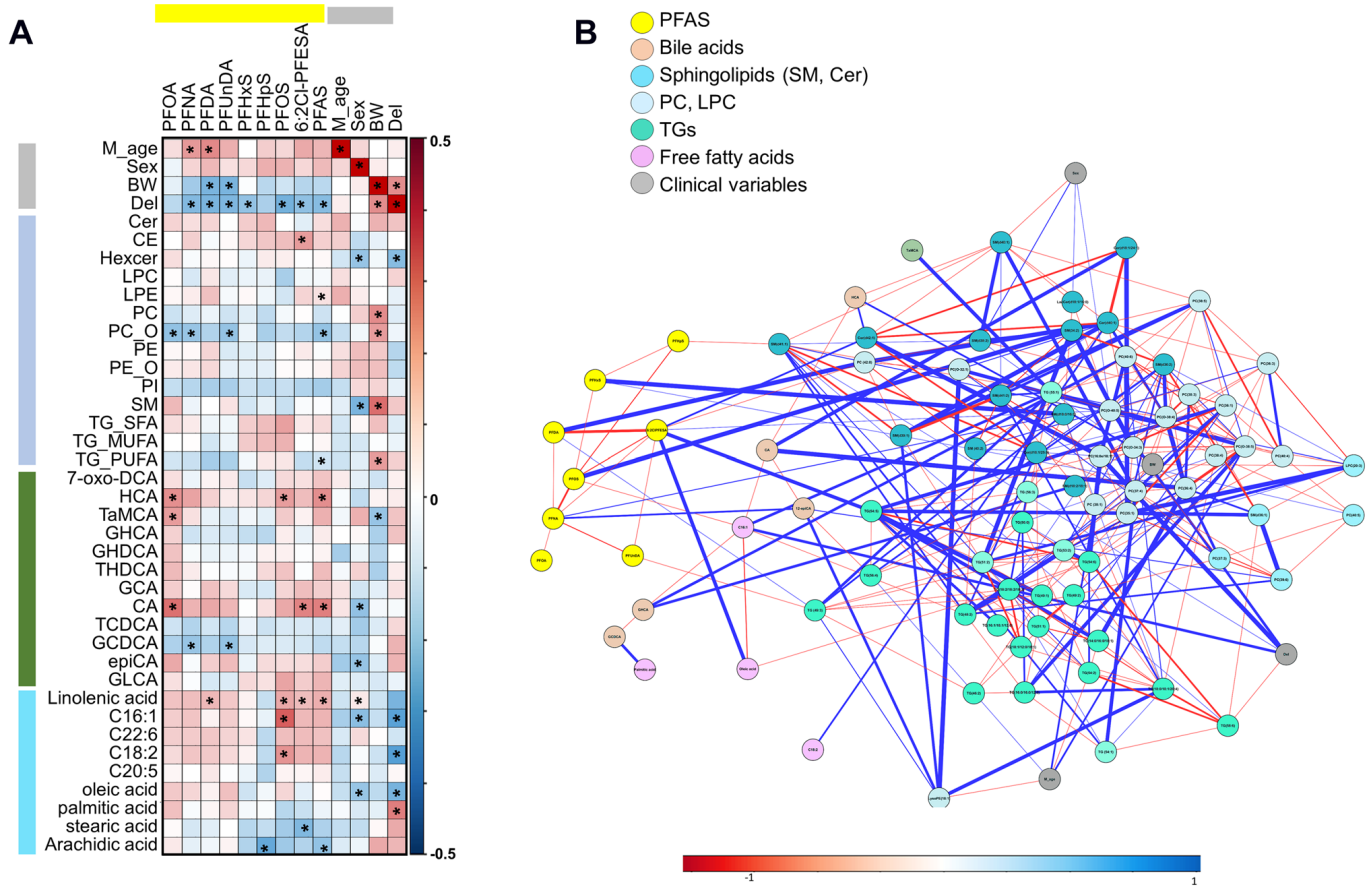
**A** Correlation plot of PFAS, bile acids, free fatty acids and lipid classes based on Spearman correlations, significant correlations (p < 0.05) marked with *. **B** Partial correlation network of the PFAS, bile acids, free fatty acids and lipids showing significant differences between the quartiles (see Table 3). Here, each node represents a compound or a clinical parameter, and edges represent the strength of partial correlation between two compounds/parameters. Edge thicknesses represent the strength of the partial correlation coefficients. Edge colors: blue color for negative correlations and red for positive correlations. Edge ranges adjusted between ± 0.25 to 1.0

Possible associations between clinical parameters, measured PFAS, lipid classes, individual lipids and BAs were studied using linear regression after adjustment with maternal age, sex and delivery type and using both Spearman correlation analysis as well as partial correlation network analysis and linear regression analysis (Fig. 2A, B, Table 2). Those individual PFAS that showed < 25% detection frequency were excluded from the statistical analyses. All PFASs were included in the total PFAS classification.

**Table 2:** Linear regression analysis across the exposure quartiles and lipids that showed significant differences, either for total PFAS as well as for individual PFAS (adjusted with maternal age, delivery and birth weight) with fold changes (FC) given as ratio of concentration between the 4th and the 1st quartiles

Lipid	FC	PFAS	PFOA	PFNA	PFHxS	PFOS	6:2 Cl-PFESA
TG (18:2/18:2/18:2)	2.07	**0.042**	0.313	0.187	0.314	0.283	0.317
TG (49:3)	1.87	**0.041**	0.908	0.639	0.103	0.471	0.643
TG (46:2)	1.81	**0.023**	0.608	0.362	0.182	0.07	0.392
TG (16:1/18:1/12:0)	1.76	**0.026**	0.34	0.105	0.072	**0.013**	0.141
TG (49:2)	1.69	**0.029**	0.577	0.133	0.131	0.148	0.375
TG (49:1)	1.68	**0.031**	0.298	0.089	0.081	0.073	0.22
TG (51:2)	1.51	**0.046**	0.639	0.248	0.197	0.34	0.41
TG (51:1)	1.5	**0.011**	0.207	**0.045**	0.094	0.064	0.125
TG (53:2)	1.43	**0.007**	0.28	0.096	0.105	0.081	0.188
TG (51:2)	1.41	**0.036**	0.555	0.196	0.231	0.209	0.318
TG (48:2	1.38	**0.05**	0.416	0.274	0.06	0.089	0.105
TG (54:5)	1.36	**0.011**	**0.008**	**0.021**	0.082	0.109	**0.026**
SM (d41:1)	1.24	**0.001**	**0.018**	**0.013**	**0.015**	**0.013**	**0.048**
C18:2	1.24	**0.029**	**0.046**	0.06	**0.04**	**0.039**	**0.04**
TG (18:2/18:2/18:2)	1.22	**0.0002**	0.124	**0.024**	**0.046**	0.084	0.11
TG (18:0/18:1/20:4)	1.2	**0.009**	0.246	0.136	0.163	0.177	0.071
TG (55:1)	1.2	**0.045**	0.241	0.094	0.108	0.14	0.073
C16:1	1.16	**0.008**	**0.013**	**0.006**	**0.021**	**0.001**	**0.001**
TG (14:0/16:0/18:1)	1.14	**0.042**	0.302	0.168	0.054	0.081	0.104
TG (54:6)	1.13	**0.017**	**0.005**	**0.016**	0.092	0.134	**0.013**
TG (50:0)	1.12	0.06	**0.044**	**0.037**	0.225	0.05	0.179
PC (40:5)	1.11	0.268	0.092	0.208	**0.017**	0.092	0.091
SM (d39:1)	1.11	**0.006**	**0.048**	0.055	**0.032**	**0.05**	0.067
TG (18:0/18:1/20:4)	1.11	**0.03**	0.294	0.207	0.352	0.514	0.212
SM(d41:2)	1.09	**0.015**	0.055	**0.045**	**0.044**	**0.016**	0.064
PC (38:1)	1.08	**0.005**	**0.005**	**0.006**	**0.009**	**0.002**	**0.007**
TG (56:4)	1.08	0.173	0.075	0.207	**0.021**	**0.01**	**0.043**
TG (56:3)	1.08	**0.036**	0.443	0.502	0.625	0.553	0.169
PC (35:1)	1.08	**0.016**	0.114	0.089	**0.018**	0.072	0.101
PC (O-38:5)	1.06	0.355	0.133	0.304	**0.027**	0.257	0.193
Oleic acid	1.06	0.096	0.109	0.133	**0.022**	0.106	0.073
PC (40:4)	1.05	**0.025**	**0.004**	**0.003**	**0.005**	**0.004**	**0.003**
PC (O-38:4)	1.04	0.668	0.165	**0.013**	0.174	0.474	**0.028**
TG (54:1)	1.04	0.322	0.358	**0.042**	0.207	0.307	0.322
PC (42:8)	1.04	0.088	**0.017**	**0.002**	0.088	0.052	**0.03**
SM (d40:1)	1.03	0.099	0.066	0.19	**0.041**	0.226	0.218
PC (O-40:5)	1.03	**0.044**	0.141	0.127	0.075	0.163	0.278
TG (58:6)	1.03	**0.002**	**0.013**	**0.003**	**0.033**	**0.023**	**0.011**
PC (40:6)	1.03	0.135	**0.046**	0.227	**0.036**	0.174	0.154
Cer (d42:1)	1.03	0.445	0.44	0.489	**0.039**	0.641	0.361
Cer (d18:1/24:1)	1.02	0.554	0.632	0.249	**0.033**	0.803	0.565
TG(54:2)	1.02	**0.041**	0.148	0.102	0.131	0.103	0.113
Cer (d40:1)	1.02	0.824	0.204	0.64	**0.024**	0.688	0.672
SM (40:2)	1.02	**0.007**	**0.021**	**0.017**	**0.021**	**0.014**	**0.03**
LacCer (d18:1/16:0)	1.02	0.188	0.064	**0.017**	0.215	0.142	0.108
PC (O-38:5)	1.01	0.084	0.066	0.133	**0.016**	0.177	0.118
PC (38:4)	1.01	**0.028**	**0.012**	**0.011**	**0.01**	**0.011**	**0.009**
TG (16:0/16:0/12:0)	1.01	0.557	0.642	0.09	0.357	**0.045**	0.063
PC (16:0e/18:1)	1.01	**0.044**	**0.048**	**0.029**	**0.023**	**0.024**	**0.018**
PC (O-34:3)	1.01	0.082	**0.043**	0.063	0.112	0.072	0.061
PC (37:3)	1.01	0.074	0.064	0.058	**0.011**	0.051	0.065
PC (O-32:1)	0.99	0.905	0.787	**0.044**	0.602	0.689	0.266
SM (d36:1)	0.99	0.065	0.076	0.092	**0.003**	0.094	0.101
PC (36:1)	0.99	**0.005**	**0.001**	**0.001**	**0.005**	**0.002**	**0.003**
SM (34:2)	0.99	**0.024**	0.219	0.11	0.157	**0.02**	0.12
PC (O-34:3)	0.98	0.903	0.32	0.736	**0.028**	0.873	0.878
SM (d36:2)	0.98	0.285	0.598	0.464	**0.012**	0.529	0.525
PC (39:6)	0.97	0.402	0.334	0.265	**0.036**	0.32	0.36
PC (38:3)	0.96	**0.01**	**0.018**	**0.029**	**0.012**	**0.018**	**0.022**
SM (d38:2)	0.96	**0.016**	**0.031**	**0.018**	**0.003**	**0.049**	**0.028**
PC (36:3)	0.96	**0.047**	0.125	0.135	0.133	0.119	0.087
PC (O-40:5)	0.94	0.061	**0.015**	**0.031**	**0.013**	**0.028**	**0.049**
PC (38:5)	0.93	**0.008**	**0.003**	**0.001**	**0.003**	**0.001**	**0.003**
PC (O-38:4)	0.92	0.153	0.114	0.249	**0.04**	0.238	0.187
PS (41:6)	0.91	**0.009**	**0.05**	**0.044**	**0.022**	**0.041**	**0.045**
PC (O-38:5)	0.91	0.149	0.193	0.468	**0.047**	0.248	0.249
palmitic acid	0.86	**0.016**	0.201	0.102	0.18	0.209	0.12
PC (37:4)	0.86	0.451	0.153	0.466	**0.016**	0.469	0.295
PC (36:4)	0.81	0.772	0.235	0.649	**0.026**	0.654	0.406
SM (d18:2/18:1)	0.79	**0.047**	**0.014**	0.071	0.255	0.329	0.106
LysoPE (18:1)	0.73	**0.022**	0.4	0.381	0.308	0.242	0.088
LPC (20:3)	0.66	**0.011**	0.556	0.57	0.296	0.371	0.284
TG (54:6)	0.39	0.072	0.123	0.091	**0.048**	0.212	0.055

Statistically significant values are highlighted in bold

Maternal age showed significant positive correlation with PFNA (R = 0.202, p = 0.04) and PFDA (R = 0.232, p = 0.02) while the correlation between maternal age with the metabolome was relatively weak with no significant correlations. Interestingly, the delivery type showed to have a significant impact on the PFAS concentrations and with hexylceramides and several free fatty acids (FFA), with significantly higher PFAS and FFA levels in cord blood of the infants that were born via C-section. Birth weight and sex of the infant showed associations with two lipid classes (sex and Hexcer R = − 0.204, p = 0.037; BW and SM R = 0.287, p = 0.003), two bile acids (sex and CA: R = − 0.213, p = 0.030; epiCA: R = − 0.204, p = 0.037), and two FFAs (sex and oleic acid: R = − 0.201, p = 0.041 and sex and C16:1: R = − 0.226, p = 0.021), p =) (Fig. 2A and B, Table 3). Next, the subjects were divided into four groups based on the total PFAS exposure quartiles (Supplementary Table 3). Using linear regression analysis, both for total PFAS as well as for individual PFAS (detection rate > 50%), significant differences between the exposure groups were observed both at the lipid classes and individual lipid levels (Table 2).

**Table 3:** Linear regression analysis between PFAS quartiles and bile acids (adjusted with maternal age, delivery, birth weight)

Dependent Variable	PFOA		PFNA		PFHxS		PFOS		6:2 Cl-PFESA		PFAS		PFOA, PFNA, PFUnDA, PFDA	
	Sig	FC	Sig	FC	Sig	FC	Sig	FC	Sig	FC	Sig	FC	Sig	FC
HCA	**0.019**	2.04	0.461	1.61	0.673	0.88	0.401	1.61	0.182	0.97	0.755	2.32	0.086	1.92
GHCA	0.443	1.14	0.129	0.96	0.557	0.87	0.066	1.03	**0.030**	1.01	**0.045**	0.96	0.511	0.96
GCA	0.330	1.11	0.728	1.17	0.687	0.93	0.158	1.17	0.365	1.05	0.297	1.07	0.714	1.13
CA	**0.004**	**1.55**	0.187	1.13	0.375	1.05	0.071	1.13	**0.016**	1.59	0.420	1.51	**0.007**	1.44
TCDCA	0.388	0.87	0.431	0.73	0.452	0.89	0.413	0.92	0.632	1.10	0.231	0.81	0.195	0.81
GCDCA	0.235	0.77	0.219	0.54	0.269	0.81	0.342	0.93	0.413	0.74	**0.011**	0.65	0.090	0.63
7-oxo-DCA	0.829	1.34	0.783	1.05	0.651	1.02	0.564	1.12	0.948	1.00	0.867	1.32	0.682	1.44
TaMCA	**0.039**	**1.44**	0.115	1.19	0.265	0.92	**0.018**	**1.16**	**0.012**	**1.05**	**0.027**	**1.19**	**0.044**	**1.39**
GHDCA	0.595	1.21	0.599	1.04	0.668	0.90	0.525	1.11	0.633	1.00	0.165	0.91	0.409	1.16
THDCA	0.383	1.20	0.741	1.35	0.626	0.75	0.174	1.15	0.689	0.99	0.819	1.16	0.529	1.16
GLCA	0.653	1.15	0.634	1.18	0.738	1.05	0.600	1.42	0.390	1.25	0.897	1.26	0.925	1.18
Total	0.514	0.84	0.428	0.75	0.392	0.78	0.297	0.96	0.470	0.88	**0.034**	0.79	0.353	0.80
Conj/unconj	0.109	0.47	0.448	0.55	0.732	0.62	0.532	0.60	0.583	0.72	0.750	0.50	*0.053*	0.51
Sec/prim	**0.005**	**1.29**	**0.039**	**1.20**	0.167	0.84	**0.030**	**1.10**	0.061	0.85	**0.024**	**1.16**	**0.001**	**1.20**
HCA_sum	0.151	1.28	0.578	1.00	0.724	0.82	0.092	1.06	0.272	0.97	0.213	1.07	0.260	1.21
HCA/CDCA	0.186	1.80	0.559	1.41	0.857	1.24	0.419	1.40	0.776	1.13	0.985	1.83	0.091	1.57
MCA/CDCA	**0.007**	**1.96**	**0.044**	**1.24**	0.190	1.42	**0.033**	**1.37**	**0.021**	**1.19**	**0.040**	**1.47**	**0.003**	**1.79**
LCA/CDCA	0.570	1.19	0.524	1.05	0.529	1.33	0.308	1.35	0.494	1.12	0.464	1.17	0.446	1.27

Statistically significant values are highlighted in bold

Fold change: Q4/Q1

Total PFAS as well as several individual PFAS showed significant strong correlation with lipids, free fatty acids, and bile acids (Fig. 2A, Table 2). Particularly, significant positive correlation was observed between cholesterol esters and 6:2 Cl-PFESA (R = 0.196, p = 0.047) and between LPE and total PFAS (R = 0.219, p = 0.025). Negative association was seen between alkylPCs and total PFAS (R = − 0.208, p = 0.034), PFOA (R = − 0.212, p = 0.030), PFNA (R = − 0.202, p = 0.040) and PFUnDA (R = − 0.207, p = 0.035), and between total PFAS and triacylglycerols with polyunsaturated fatty acyls (R = − 0.214, p = 0.029). PFOS showed negative correlation with two saturated fatty acid (stearic acid: R = − 0.248, p = 0.011 and arachidic acid: R = − 0.301, p = 0.0019). PFHpS and total PFAS showed positive correlation with monounsaturated palmitoleic acid (R = 0.316, p = 0.0011) and 6:2Cl-PFESA with linoleic acid (R = 0.212, p = 0.030). Most of the individual PFASs showed similar impacts on lipids. The individual lipids showed, as expected, similar association with the exposure. Three bile acids were positively correlated with PFOA (HCA: R = 0.220, p = 0.025, TaMCA: R = 0.193, p = 0.0049 and CA: R = 0.285, p = 0.004), one with PFOS (HCA, R = 0.194, p = 0.049) and 6:2Cl-PFESA (R = 0.244, p = 0.012). Also total PFAS showed positive association with HCA (R = 0.223, p = 0.023) and CA (R = 0.252, p = 0.0098) while PFNA and PFUnDA showed negative association with GCDCA (R = − 0.202, p = 0.040 and R = − 0.209, p = 0.033, respectively). The partial correlation network analysis of those lipids that showed significant differences in liner regression analysis showed that in addition to chemical class specific interactions, individual PFAS were linked with multiple lipids and specific bile acids and also with some free fatty acids while lipids and bile acids showed strong interactions with each other (Fig. 2B). Clinical parameters (sex, maternal age, birth weight, delivery) were associated with multiple lipids, mainly TGs.

**Fig. 3:**
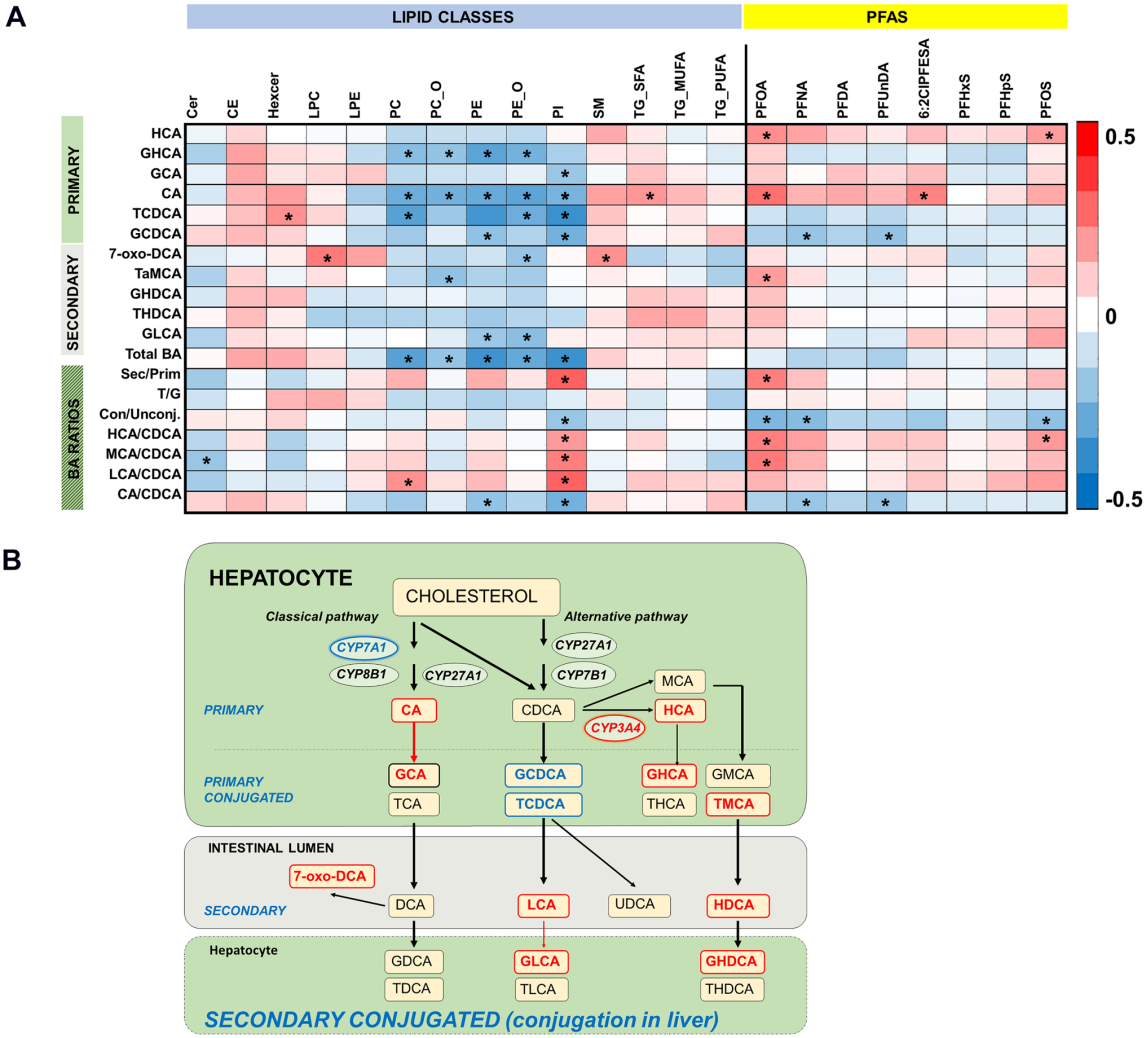
**A** Spearman correlations of bile acids, indicative bile acid ratios, lipids and PFAS. Significant correlations marked with asterix. **B** An overview of bile acid biosynthesis pathways. Bile acids detected in cord blood marked with bold text, upregulated by PFAS marked with red, down-regulated with blue. Bile acids are synthesized from cholesterol via two pathways. The major, classic pathway is initiated and rate-limited by CYP7A1 to synthesize CA and CDCA, regulated further by CYP8B1 and CYP27A1. The alternative pathway is initiated and catalyzed by CYP27A1 to synthesize CDCA. CDCA can be further converted to HCA by CYP3A4 and MCA in the liver. Then the BAs are conjugated to the amino acids taurine or glycine before being released into the intestine. In the distal ileum and colon, the BAs undergo a variety of bacterial transformations including deconjugation, dehydroxylation, and epimerization by the gut microbiota producing a variety of secondary bile acids. In humans, HCA and MCA can potentially be produced also by gut microbiota. During the enterohepatic circulation, the secondary BAs are conjugated in the liver

Next, we investigated the PFAS-lipid-bile acid associations in more detailed level, including specific bile acid ratios that are indicative of the bile acid synthesis pathways (Fig. 3, Table 3). CA which was positively associated with PFOA and 6:2Cl-PFESA (R > 0.244, p < 0.012) showed significant negative association with phospholipids (PC, PC_O, PE, PE_O and PI with R <) and positive association with TGs with saturated fatty acids. Interestingly, the two conjugates of the primary bile acid CDCA showed different trend (Table 3, Fig. 3): GCDCA was negatively associated both with PFNA and PFUnDA (R < − 0.202, p < 0.040) and both GCDCA and TCDCA were further positively associated with PEs and PIs (R between − 0.215 and − 0.366, and p < between 5.7 × 10^–5^ and 0.028). Overall, PFAS with carboxylic acid moiety had a more pronounced impact on the bile acids as compared with the PFAS with a sulfuric acid moiety. Overall, the total bile acids and several individual bile acids were negatively associated with PCs, PEs and PI while multiple bile acids showed positive association with Hexcers and LPCs and SM. The total pool of the bile acids did not show significant association with the PFAS, however, there was significant associations with the bile acid conjugation ratio as well as in the ratio of CDCA and the bile acids derived from CDCA (HCA, MCA) as well as in CA/CDCA ratio.

## Discussion

Our study explored the effects of in utero exposure to PFAS on fetal metabolome by assessing the associations between PFAS, lipids and bile acids in cord blood. Overall, the PFAS levels measured in the cord blood in this study were at similar levels than what has been reported in other studies (Richterová et al., 2018; Stock et al., 2007; Workman et al., 2019). One important observation is that 6:2 Cl-PFESA has been detected only in the Chinese population, as this compound is only produced and has been used in China to replace PFOS. This substance is of great concern, as it has been shown to cross the placenta and enter the fetal compartment more readily than *e.g.* PFOS (Pan et al., 2017).

The main finding of the current study was the significant associations observed between the PFAS, lipids and bile acids. We observed that particularly those PFAS that had a carboxylic acid moiety were associated with up-regulation of bile acids in the cord blood, with the two CDCA conjugates being an exception, being significantly down-regulated. In adults, PFAS exposure suppresses the biosynthesis of bile acids through suppression of the CYP7A1 in the liver (Beggs et al., 2016; Chiang, 2017). However, in fetus, opposite to the postnatal life, the biosynthesis of bile acids is utilizing mainly the secondary, acidic pathway, regulated by sterol 27-hydroxylase which has not been shown to be affected by PFAS exposure and with CDCA as the main BA synthesised (Chiang, 2017; Sigurdsson et al., 2016). While we did observe that PFAS exposure was associated with decreased levels of CDCA conjugates, there was an increase of the CDCA derived bile acids (HCA, MCA and their conjugates, and the secondary BAs derived from these, LCA and HDCA). Specifically, we observed up-regulation of HCA/CDCA ratio, indicating that more HCA was produced from the CDCA. This pathway is regulated by CYP3A4 which in human hepatocytes have shown to be up-regulated by PFAS (Louisse et al., 2020) thus supporting our results. Overall, in fetus, PFAS does not seem to have a major suppressing effect on the bile acid biosynthesis, as we observed positive association between the majority of the bile acids and PFAS. Our data agrees with the findings of the animal models that showed increased total bile acid concentrations in cord blood due to PFAS exposure, potentially due to decreased levels of BAs in the liver, reflecting to less efficient extraction of bile acids from the sinusoidal blood to the liver due to PFAS exposure. Our data thus indicates that in human fetus, the PFAS exposure disturbs the BA metabolism by a distinctively different mechanism than in the adults; however, more studies are required for verification of these findings.

Consistent with other human studies on PFAS (McGlinchey et al., 2019; Salihovic et al., 2018; Salihović et al., 2019; Sinisalu et al., 2020), we found that plasma PFAS concentrations were significantly associated with dysregulation of multiple lipid classes. Importantly, PFAS exposure was associated with increased levels of TGs with saturated/monounsaturated fatty acids, and decreased levels of several phospholipids and PUFA containing TGs. Similar changes due to PFAS exposure have been reported in animal models (Adinehzadeh & Reo, 1998; Bijland et al., 2011b). Alteration of these types of metabolites has also reported to be associated with type 2 diabetes, NAFLD and in obesity (Hyotylainen et al., 2016; Oresic et al., 2013). One suggested mechanism of PFAS exposure is an inhibition of mitochondrial fatty acid β-oxidation in which fatty acids are diverted to TG synthesis (Begriche et al., 2013; Das et al., 2017; Nault et al., 2017; Yu et al., 2016). We observed significant up-regulation of TGs fatty acyls shorter than 18 carbons, and with low number of double bonds, thus indicating suppression of mitochondrial fatty acid β-oxidation, in a similar manner that we have observed earlier in an animal model using avian embryos (Geng et al., 2019). The impact of PFAS on lipid metabolism may also be modulated through the bile acids, as the bile acid-activated farnesoid × receptor (FXR) has also a regulatory role in triglyceride metabolism. In the liver, FXR activation results in down-regulation of CYP7A1, which in addition to inhibition of the classical bile acid synthetic pathway also reduces the expression of several genes mediating free fatty acid synthesis, thereby attenuating de novo lipogenesis (Chiang, 2017; Honda et al., 2019; Ticho et al., 2019). Thus, activation of FXR modulates FFA oxidation and triglyceride clearance to the circulation. At organism level, it has been suggested that exposure to PFAS increases steatosis, i.e. hepatic accumulation of lipids, because the balance of fatty acid accumulation/synthesis and oxidation is disrupted to favor accumulation (Jin et al., 2020).

The significant impact of PFAS exposure on bile acids and TGs may cause significant adverse impacts on the fetus. Studies of intrahepatic cholestasis of pregnancy (ICP), where the bile acids are elevated in the mother, have shown that the rates of fetal complications are positively correlated with the total level of bile acids in maternal serum (Glantz et al., 2004). In ICP, the fetal–maternal bile acid gradient is inverted, and the bile acid transfer from fetus-to-mother is also impaired, thus contributing to an accumulation of bile acids in the fetal compartment (Serrano et al., 1998). ICP is associated with an increased risk of adverse fetal outcomes, including spontaneous preterm labour, meconium staining of the amniotic fluid, low Apgar scores, and sudden intrauterine death (Glantz et al., 2004). As PFAS exposure in our current study was showing similar changes, i.e. increment of the bile acids levels in fetus, the health impacts may also be similar. Moreover, we have observed similar changes in our previous prospective studies further supporting our findings (Schlezinger et al., 2020; Sinisalu et al., 2020).

Elevated TG levels at birth have been significantly linked with children’s psychological and social functioning 5 years later, suggesting that fetal exposure to lipids may represent a novel form of biological embedding for psychological health risk (Manczak & Gotlib, 2019). Moreover, increased levels of TGs with saturated or monounsaturated fatty acyls we observed in our study have been linked with non-alcoholic fatty liver disease (NAFLD) as well as insulin resistance in adults (Oresic et al., 2013). Indeed, it has been suggested that onset of NAFLD in obese children and adults may have fetal origins (Baker & Friedman, 2018). Particularly, it has been implicated that increased hepatic lipids, disrupted mitochondria, and elevated oxidative stress, along with increased de novo lipogenesis in fetus may contribute to lifetime risk of NAFLD and likely contribute to the severity and early onset of NAFLD in children (Baker and Friedman, 2018).

The present study has several strengths. First, this study employed a comprehensive analysis of lipid profiles and PFAS in the cord blood, allowing us to study how exposure to mixtures of PFAS may affect the fetal metabolome. The study also had some limitations. First, results are correlative in nature and from a small sample size and all lifestyle factors were not available. Thus, some of the possibly confounding factors may be related to differences in the lifestyle factors, such as maternal diet or smoking. However, in our previous studies we have not observed any major association between the maternal diet or smoking and lipidome in cord blood (McGlinchey et al., 2019). Also, due to the cross-sectional study design, the temporal relationship between PFAS and lipids cannot be assured. However, majority of PFAS have a long half-life in blood and therefore, their levels are likely to reflect the fetal exposure. All in all, future work is needed to elucidate the mechanisms involved.

## Conclusions

Taken together, our results indicate that prenatal PFAS exposure may modulate and affect lipid and BA metabolism in concentration-dependent manner. Importantly, PFAS exposure was associated with significant increase of those lipids that have been linked with several adverse health conditions later in life, such as type 2 diabetes and NAFLD. Our study thus underscores the need for investigation of how exposure to specific PFAS and other persistent chemical pollutants during pregnancy and early childhood affect the risk and pathogenesis of these diseases.

## Supplementary Information

Below is the link to the electronic supplementary material.

Supplementary file1 (DOCX 27 KB)

## Data Availability

The metabolomics and metadata reported in this paper are available on request due to privacy/ethical restrictions.
