# Supplementary material for: Prenatal exposure to poly-/per-fluoroalkyl substances is associated with alteration of lipid profiles in cord-blood
Source: Metabolomics. 2021 Nov 24;17(12):103. doi: 10.1007/s11306-021-01853-9 (PMC8610959; doi:10.1007/s11306-021-01853-9)
Supplement: Supplementary file 1 — Supplementary file1 (DOCX 27 KB) [file 11306_2021_1853_MOESM1_ESM.docx]

**Supplementary data**

**Content:**

**Supplementary tables:**

**Supplementary Table 1.** Abbreviations of the PFAS, bile acids, lipids and others.

**Supplementary table 2.** PFAS exposure groups.

**Supplementary Table 1.** Abbreviations of the PFAS, bile acids and lipids.

| **Abbreviation** | **Compound** | **Purchased from** |
| --- | --- | --- |
| 12-epiCA | 3α, 7α, 12β-trihydroxy-5β-cholan-24-oic acid |  |
| 4:2FTSA | 4:2 fluorotelomer sulfonic acid | synthesized |
| 6:2 Cl-PFESA | 6:2 chlorinated polyfluoroalkyl ether sulfonic acid | Wellington Laboratories |
| 6:2FTSA | 6:2 fluorotelomer sulfonic acid | synthesized |
| 7-oxo-DCA | 7-oxodeoxycholic acid | Steraloids |
| 8:2 Cl-PFESA | 8:2 chlorinated polyfluoroalkyl ether sulfonic acid | Wellington Laboratories |
| 8:2FTSA | 8:2 fluorotelomer sulfonic acid | Wellington Laboratories |
| C16:1 | Palmitoleic acid | Sigma-Aldrich |
| C18:2 | Linoleic acid | Sigma-Aldrich |
| C20:5 | Eicosapentaenoic acid | Sigma-Aldrich |
| C22:6 | Cervonic acid | Sigma-Aldrich |
| CA | Cholic acid | Sigma-Aldrich |
| CDCA | Chenodeoxycholic acid | Sigma-Aldrich |
| GCA | Glycocholic acid | Sigma-Aldrich |
| GCDCA | Glycochenodeoxycholic acid | Sigma-Aldrich |
| GHCA | Glycohyocholic acid | Steraloids |
| GHDCA | Glycohyodeoxycholic acid | Steraloids |
| GLCA | Glycolithocholic acid | Calbiochem |
| PFAS | Per- and Polyfluoroalkyl substances | Wellington Laboratories |
| PFBA | Perfluorobutanoic acid | Wellington Laboratories |
| PFBS | Perfluorobutane sulfonic acid | Wellington Laboratories |
| PFDA | Perfluorodecanoic acid | Wellington Laboratories |
| PFDoDA | Perfluorododecanoic acid | Wellington Laboratories |
| PFDoDS | Perfluorododecane sulfonic acid | Wellington Laboratories |
| PFDS | Perfluorodecane sulfonic acid | Wellington Laboratories |
| PFHpA | Perfluoroheptanoic acid | Wellington Laboratories |
| PFHpS | Perfluoroheptane sulfonic acid | Wellington Laboratories |
| PFHxA | Perfluorohexanoic acid | Wellington Laboratories |
| PFHxDA | Perfluorohexadecanoic acid | Wellington Laboratories |
| PFHxS | Perfluorohexane sulfonic acid | Wellington Laboratories |
| PFNA | Perfluorononanoic acid | Wellington Laboratories |
| PFNS | Perfluorononane sulfonic acid | Wellington Laboratories |
| PFOA | Perfluorooctanoic acid | Wellington Laboratories |
| PFOS | Perfluorooctane sulfonic acid | Wellington Laboratories |
| PFPeA | Perfluoropentanoic acid | Wellington Laboratories |
| PFPeS | Perfluoropentane sulfonic acid | Wellington Laboratories |
| PFTDA | Perfluorotetradecanoic acid | Wellington Laboratories |
| PFTeDA | Perfluorotetradecanoic acid | Wellington Laboratories |
| PFTriDA | Perfluorotridecanoic acid | Wellington Laboratories |
| PFUnDA | Perfluoroundecanoic acid | Wellington Laboratories |
| TCA | Taurocholic acid | Sigma-Aldrich |
| TCDCA | Taurochenodeoxycholic acid | Sigma-Aldrich |
| TDCA | Taurodeoxycholic acid | Sigma-Aldrich |
| TDHCA | Taurohyodeoxycholic acid | Sigma-Aldrich |
| THCA | Taurodeoxycholic acid | Sigma-Aldrich |
| THDCA | Taurohyodeoxycholic acid | Sigma-Aldrich |
| TαMCA | α-tauromuricholic acid | Steraloids |
| CE (17:0) | Cholesteryl heptadecanoate | Larodan |
| CE(16:0) | Cholesteryl palmitate | Larodan |
| CE(18:1) | Cholesteryl linoleate | Larodan |
| Cer(d18:1/17:0) | N-(heptadecanoyl)-sphing-4-enine | Avanti |
| Cer(d18:1/18:1) | N-Octadecenoyl-(cis-9)-D-erythro-Sphingosine | Avanti |
| DG(18:1/18:1) | 1-2-dioleoyl-sn-glycerol | Larodan |
| LysoPC(17:0) | 1-heptadecanoyl-sn-glycero-3-phosphocholine | Avanti |
| LysoPC(18:0) | 1-octadecanoyl-sn-glycero-3-phosphocholine | Avanti |
| LysoPC(18:1) | 1-octadecenoyl-sn-glycero-3-phosphocholine | Avanti |
| LysoPE(18:1) | 1-(octadecenoyl)-sn-glycero-3-phosphoethanolamine | Avanti |
| PC(16:0/16:0) | 1,2-dihexadecanoyl-sn-glycero-3-phosphocholine | Avanti |
| PC(16:0/18:1) | 1-hexadecanoyl-2-(octadecenoyl)-sn-glycero-3-phosphocholine | Avanti |
| PC(16:0d31/18:1) | 1-hexadecanoyl -d31-2-(9Z-octadecenoyl)-sn-glycero-3-phosphocholine | Avanti |
| PC(17:0)/17:0) | 1,2-diheptaadecanoyl-sn-glycero-3-phosphocholine | Avanti |
| PC(18:0/18:0) | 1,2-dioctadecanoyl-sn-glycero-3-phosphocholine | Avanti |
| PE(16:0/18:1) | 1-hexadecanoyl-2-(octadecenoyl)-sn-glycero-3-phosphoethanolamine | Avanti |
| PE(17:0/17:0) | 1,2-(heptadecenoyl)-sn-glycero-3-phosphoethanolamine | Avanti |
| SM(d18:1/17:0) | N-heptadecanoyl-D-erythro-sphingosylphosphorylcholine | Avanti |
| SM(d18:1/18:1) | N-octadecenoyl-D-erythro-sphingosylphosphorylcholine | Avanti |
| TG(16:0/16:0/16:0) | Tripalmitin | Avanti |
| TG(17:0/17:0/17:0) | Triheptadecanoin | Larodan |
| TG(18:0/18:0/18:0) | Trioctadecanoin | Larodan |

**Supplementary table 2.** PFAS exposure groups.

|  | **Quartile 1** | **Quartile 2** | **Quartile 3** | **Quartile 4** |
| --- | --- | --- | --- | --- |
|  | **(ng/ml, min-max)** | **(ng/ml, min-max)** | **(ng/ml, min-max)** | **(ng/ml, min-max)** |
| **PFAS** | 0.67-1.16 | 1.18-1.71 | 1.73-2.35 | 2.37-5.4 |
| **PFOA** | 0.36-0.49 | 0.52-0.66 | 0.66-0.87 | 0.88-3.6 |
| **PFNA** | LOD-0.65 | 0.65-0.84 | 0.84-0.12 | 0.12-0.31 |
| **PFHxS** | LOQ-0.06 | 0.07-0.09 | 0.09-0.14 | 0.14-2.1 |
| **PFOS** | LOQ-0.28 | 0.28-0.45 | 0.045-0.70 | 0.70-1.80 |
| **6:2ClPFESA** | 0.07-0.13 | 0.13-0.19 | 0.19-0.27 | 0.27-1.82 |

**Supplementary table 3.** Detection frequency (%) and PFAS concentration (ng/mL) in cord serum samples from different studies

|  |  | **PFOA** | **PFNA** | **PFHxS** | **PFOS** | **Reference** |
| --- | --- | --- | --- | --- | --- | --- |
| Beijing (*n=100*), 2018 | | | | | | Current investigation |
| Detection frequency (%) | | 100 | 89 | 99 | 99 |  |
| Median (ng/mL) | | 0.661 | 0.084 | 0.089 | 0.449 |  |
| Slovakia (*n=322*), 2010–2012 | | | | | | Richterova et al., 2018 |
| Detection frequency (%) | | 99.7 | 99.4 | 93.1 | 100 |  |
| Median (ng/mL) | | 0.88 | 0.24 | 0.008 | 0.64 |  |
| Winnipeg, Canada (*n=50*), 2010–2011 | | | | | | Workman et al., 2019 |
| Detection frequency (%) | | 100 | 34 | 82 | 100 |  |
| Median (ng/mL) | | 0.58 | <LOQ | 0.26 | 0.8 |  |
| South Korea (*n=43*), 2008–2009 | | | | | | Kim et al., 2011 |
| Detection frequency (%) | | 100 | 63 | 100 | 100 |  |
| Median (ng/mL) | | 1.15 | 0.45 | 0.34 | 1.26 |  |
